# A case of long-term survival after surgical resection for solitary adrenal recurrence of esophageal squamous carcinoma

**DOI:** 10.1186/s40792-017-0337-8

**Published:** 2017-05-05

**Authors:** Nobuhiko Kanaya, Kazuhiro Noma, Tsuyoshi Okada, Naoaki Maeda, Shunsuke Tanabe, Kazufumi Sakurama, Yasuhiro Shirakawa, Toshiyoshi Fujiwara

**Affiliations:** 1 Department of Gastroenterological Surgery, Okayama University Graduate School of Medicine, Dentistry and Pharmaceutical Sciences, 2-5-1 Shikata-cho, Kita-ku, Okayama, 700-8558 Japan; 2 Department of Surgery, Shigei Medical Research Institute, Okayama, Japan

**Keywords:** Esophagectomy, Adrenal metastasis, Esophageal squamous cell carcinoma

## Abstract

**Background:**

Esophageal carcinomas are highly malignant tumors with a high frequency of lymph node and distant organ metastasis. Treatment for recurrent tumors is generally decided on an individual basis. Although multidisciplinary treatments involving chemotherapy, surgical resection, and radiation are performed, the prognosis remains poor. Here, we report a case of prolonged recurrence-free survival (38 months) after esophageal carcinoma surgery and subsequent laparoscopic adrenalectomy for right adrenal metastasis.

**Case presentation:**

An 83-year-old man was diagnosed with type 3 esophageal squamous cell carcinoma (T3N1M0, cStage IIIA, UICC-7), spreading from the lower thoracic esophagus to the abdominal esophagus. He underwent thoracoscopic esophagectomy with a two-field lymph node dissection followed by substernal gastric tube reconstruction. The final diagnosis was moderately differentiated squamous cell carcinoma (T3N2M0, fStage IIIB). Adjuvant chemotherapy was not administered because of the advanced age and postoperative condition of the patient. Computed tomography (CT) at 14 months postoperatively showed a mass with a 2-cm diameter at the right adrenal gland. Positron emission tomography (PET)/CT revealed a high fluorodeoxyglucose (FDG) uptake in the mass. It was suspected that the mass was a metastatic lesion secondary to the primary esophageal carcinoma. No metastases to lymph nodes or other distant organs were identified. The patient underwent laparoscopic right adrenalectomy. The histopathological examination revealed moderately differentiated squamous cell carcinoma, suggesting metastasis from the primary esophageal carcinoma. He has survived without recurrence for 38 months since laparoscopic adrenalectomy to remove the right adrenal metastastic mass after the esophageal carcinoma surgery.

**Conclusions:**

We describe a very elderly male who survived laparoadrenalectomy for right adrenal metastasis following esophageal cancer surgery without recurrence for 38 months postoperatively. Therefore, surgical resection might be an option for solitary adrenal recurrence.

## Background

Esophageal carcinomas are highly biologically malignant tumors because the high frequency of lymph node and distant organ metastasis [1]. Common patterns of recurrence are metastasis to the lymph nodes, lungs, liver, bone, brain, and adrenal glands [2, 3]. Treatment for recurrent lesions is generally decided on an individual basis. Although multidisciplinary treatments are combined with chemotherapy, surgical resection, and radiation, the prognosis remains poor [4, 5]. The role of surgical resection for metastasis from esophageal carcinoma has not been clarified, but some reports have recently described the benefit of resection for oligometastasis from esophageal carcinoma [6, 7].

Here, we report a case of a 38-month recurrence-free survival after laparoscopic adrenalectomy for right adrenal metastasis after esophageal carcinoma surgery.

## Case presentation

**Fig. 1:**
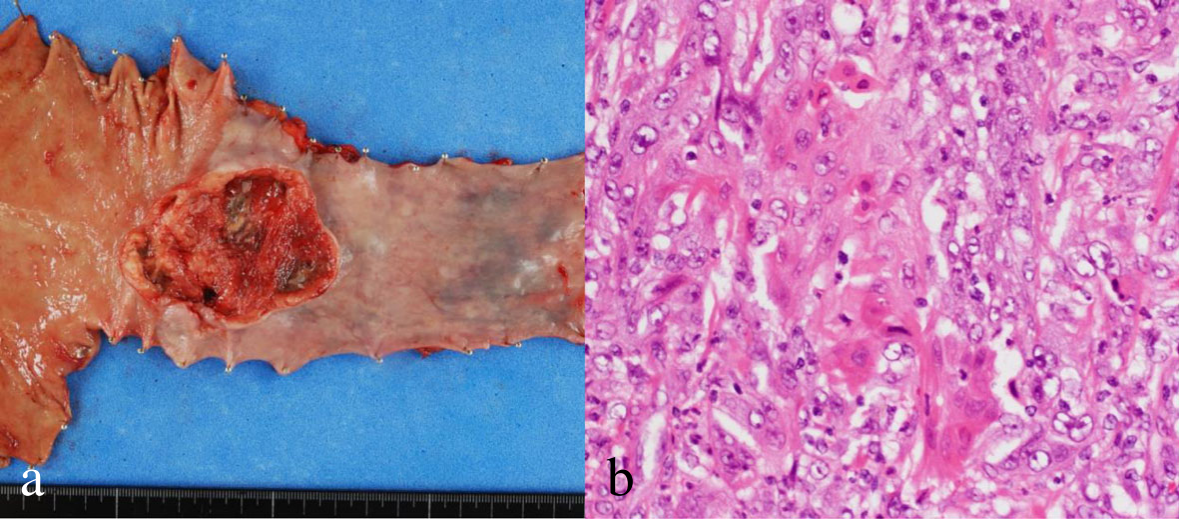
Resected specimen of esophageal carcinoma. **a** Gross appearance shows type 3 tumor, approximately 4.5-cm long in LtAe. **b** Hematoxylin and eosin staining shows moderately differentiated squamous cell carcinoma

An 83-year-old man was diagnosed with type 3 esophageal squamous cell carcinoma (LtAe, T3N1M0, cStage IIIA, UICC-7) measuring 6 cm in diameter. Computed tomography (CT) and 8F-fluorodeoxyglucose (FDG) positron emission tomography (PET)/CT showed no metastasis to distant organs, whereas metastatic lymph nodes were present at the lesser curvature of the stomach. The patient underwent thoracoscopic esophagectomy with two-field lymph node dissection, laparoscopic substernal gastric tube reconstruction (Fig. 1). The final diagnosis was moderately differentiated squamous cell carcinoma (T3N2M0, stage IIIB). Three lymph nodes at the lesser curvature of the stomach were diagnosed as containing metastatic squamous cell carcinoma. The patient suffered from diarrhea after the operation. Adjuvant chemotherapy was not administered because of the patient’s advancing age and postoperative condition.

**Fig. 2:**
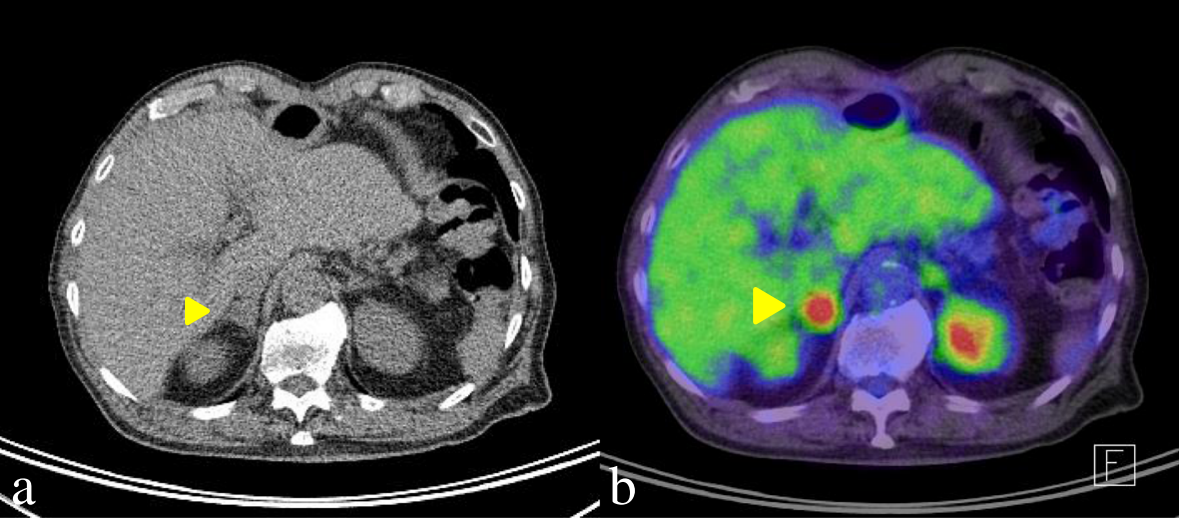
Images from CT and FDG-PET/CT. **a** A mass with a 2-cm diameter in the right adrenal gland. **b** PET/CT shows high accumulation of FDG (standardized uptake value max, 7.67) in the right adrenal mass. Abbreviations: *CT* computed tomography, *PET* positron emission tomography, *FDG* 8F-fluorodeoxyglucose

**Fig. 3:**
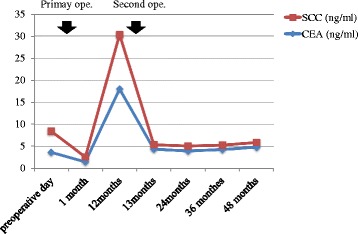
Clinical changes in concentrations of tumor markers, carcinoembryonic antigen (*blue line*) and squamous cell carcinoma (*red line*). *Ope* operation

**Fig. 4:**
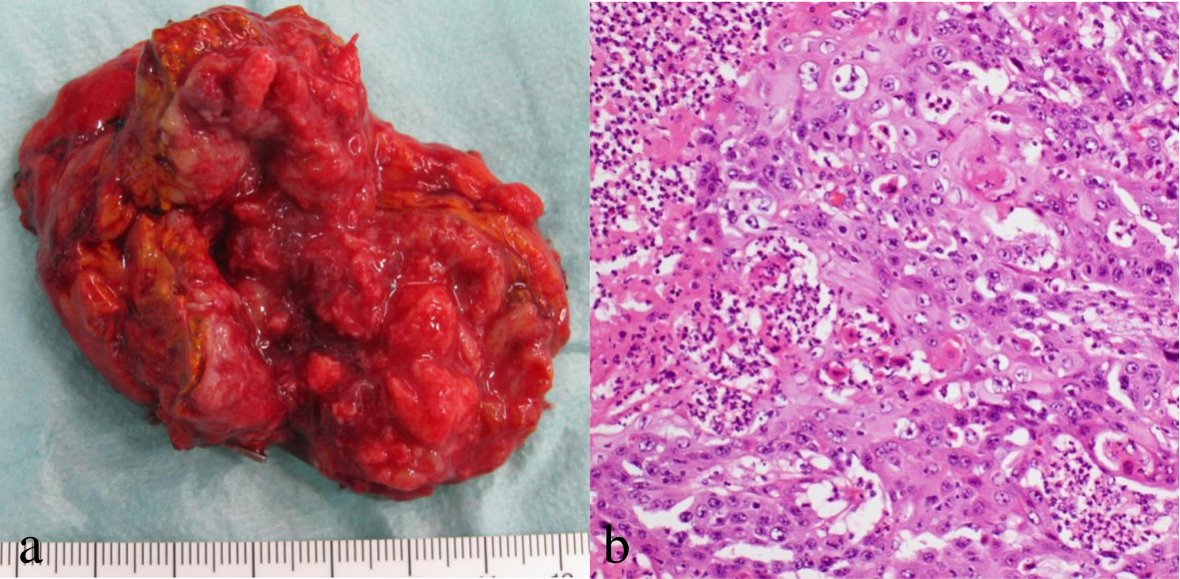
Resected specimen of the right adrenal mass. **a** Gross appearance shows a hard, solid mass approximately 2.0 cm in diameter. **b** Hematoxylin eosin staining shows moderately differentiated squamous cell carcinoma

After treatment, follow-up was conducted on an outpatient basis once every 3 months. Follow-up included physical examination and laboratory tests, including those for tumor markers. Alternating 6-month periods of upper gastrointestinal endoscopy, contrast-enhanced CT, and FDG-PET/CT were performed. CT at 14 months postoperatively showed a small growing mass, which had enlarged to 2 cm in diameter in the right adrenal gland (Fig. 2) and PET/CT revealed high FDG uptake at the mass (maximum standardized uptake value (SUV), 7.67). The physical examination at that time showed no abnormalities. Laboratory tests showed that concentrations of squamous cell carcinoma (SCC) antigen and carcinoembryonic antigen (CEA) had increased to 12.3 ng/ml (normal range, 0–1.5 ng/ml) and 18.01 ng/ml (normal range, 0–5.0 ng/ml), although these concentrations had been normal just after the primary operation (Fig. 3). The mass was suspected to be a metastasis from the primary esophageal carcinoma. No metastases to lymph nodes or other distant organs were apparent. The patient was admitted to our hospital for surgery and underwent laparoscopic right adrenalectomy from the left lateral decubitus position. A port for endoscopy was present where the right arcus costalis and external marginal abdominis muscle crossed and the other three ports were along the subcostal lines. If there had been severe intra-abdominal adhesion, then the laparoscopic approach would have been changed to the retroperitoneal approach. Though there was inflammatory adhesion between the inferior vena cava and adipose tissue around the tumor, it was possible to exfoliate them. This second operation ended without any problems because the right adrenal gland was anatomically away from the gastric tube. The operative time was 127 min. The volume of blood loss was 30 ml. The histopathological examination revealed moderately differentiated squamous cell carcinoma, strongly suggesting the presence of metastasis from the primary esophageal cancer (Fig. 4). Laboratory tests after this second operation showed decreased concentrations of SCC antigen and CEA to 1 and 4.4 ng/ml, respectively. Thus far, no postoperative recurrences have occurred as of 38 months after adrenalectomy.

## Conclusions

Distant recurrence after curative esophageal carcinoma surgery is one of the most difficult complications to treat. Recently, multidisciplinary therapy has been performed for recurrent esophageal carcinoma. However, the prognosis of the patients with recurrence is very poor, with a reported survival of 4–7 months [8, 9].

The benefit of surgical resection for patients with recurrence remains controversial. Some reports indicated that lymphadenectomy or chemoradiotherapy might improve survival in patients with lymph node recurrence in the neck or mediastinum after curative resection [4, 5]. Patients with lung metastasis reportedly show relatively good prognosis, with a median survival of 9.8 months [8].Whereas, patients with liver metastasis reportedly exhibit poor prognosis because of the high frequency of multiple metastases [6]. The initial recurrent site could thus contribute to the prognostic heterogeneity of the patients with recurrent esophageal carcinoma.

Most tumors arising in the adrenal gland are benign adenomas [10], and malignant tumors of the adrenal gland are quite rare. The frequency of non-adrenal carcinoma metastases to the adrenal gland is of approximately 0.7–2.5% [10]. Common carcinomas associated with metastases to the adrenal glands are lung, gastrointestinal, breast, kidney carcinoma, and melanoma. Adrenal metastasis is generally identified on CT, PET, or MRI, during postoperative follow-up [10, 11]. Therapy for adrenal metastasis is decided on an individual basis depending on the primary carcinoma. The characteristics of adrenal metastasis on imaging studies include an irregular shape, inhomogeneous nature, high vascularity on contrast-enhanced CT, and elevated SUV on PET [11]. In our case, the adrenal mass was found on a follow-up CT and FDG-PET/CT in the absence of other primary lesions. Tumor markers such as SCC and CEA were also elevated. Additionally, FDG uptake was high despite the relatively small size. Metastasis from esophageal carcinoma was therefore suspected.

Adrenal metastasis in patients with esophageal carcinoma is very rare [12]. We found only eight cases between 1995 and 2015 (Table 1) [13–18]. The sex ratio showed a clear male predominance (male:female, 8:0). Median age at primary diagnosis was 63 years (range, 50–83 years). The adrenal metastasis was right-sided in three patients and left-sided in five. The median interval from the primary operation to discovery of metastasis was 8 months. Patients who underwent adrenalectomy survived for a median of 36 months (range, 14–71 months), with all achieving relatively long survival after adrenalectomy. Thus, in our very elderly patient, laparoscopic adrenalectomy was selected.

**Table 1:** Eight patients with surgical resection of adrenal metastasis from esophageal carcinoma

Year	Author	Age	Sex	Location of the EC	Histology	fStage	Location of the AM	Size of AM (cm)	Interval from the EC	Prognosis	Chemotherapy
1992	Shimada	59	Male	MtLt	SCC	III	Right	6 × 6 cm	4 months	18 months alive	Unknown
1997	Yoshizumi	56	Male	MtLt	SCC	IV	Left	1.5 × 1.5 cm	0	22 months alive	+
1997	Hata	67	Male	MtLt	SCC	III	Left	6.5 × 5.5 cm	8 months	14 months alive	−
2004	Nagano	57	Male	MtLt	SCC	II	Left	6.2 × 4.8 cm	3 months	unknown	Unknown
2006	MM.Cho	70	Male	LtAe	SCC	III	Left	5 × 4 cm	8 months	42 months alive	+
2010	Saito	71	Male	Mt	Adeno	III	Right	2.5 × 2 cm	22 months	71 months alive	+
2013	O’Sullivan KE	50	Male	AeG	Adeno	II	Left	Unknown	48 months	48 months alive	+
2016	Our case	83	Male	LtAe	SCC	III	Right	2 cm	14 months	36 months alive	−

*SCC* squamous cell carcinoma, *Adeno* adenocarcinoma, *AM* adrenal metastasis, *EC* esophageal carcinoma

In general, chemotherapy is chosen for distant recurrence of esophageal carcinoma because of the oncological systemic status. Chemotherapy such as S-1 was considered for this patient. However, chemotherapy was not recommended for our patient because of his advanced age and concern about the side effects such as diarrhea. Thus, with sufficiently informed consent, surgical resection represents an option for solitary adrenal metastasis, even for those with distant recurrence. Best practice therapy including surgical resection and radiation therapy as well as chemotherapy is thus important to consider for patients with adrenal metastasis recurrence of esophageal carcinoma. In particular, surgical resection might be more important for solitary adrenal metastasis than for other distant metastases.

We report a case of prolonged recurrence-free survival (38 months) after laparoadrenalectomy for right adrenal metastasis following esophageal cancer surgery in a very elderly male. Surgical resection might be an option for solitary adrenal recurrence.

## Abbreviations

CEA: Carcinoembryonic antigen
CT: Computed tomography
FDG: 8F-fluorodeoxyglucose
PET: Positron emission tomography
SCC: Squamous cell carcinoma
SUV: Standardized uptake value
